# Dermatofibrosarcoma protuberans with fibrosarcomatous transformation of the head and neck

**DOI:** 10.1186/1758-3284-3-5

**Published:** 2011-02-04

**Authors:** Nikolaos Angouridakis, Panagiotis Kafas, Waseem Jerjes, Stefanos Triaridis, Tahwinder Upile, Georgios Karkavelas, Angelos Nikolaou

**Affiliations:** 1 Otorhinolaryngology, Head and Neck Surgery Department, AHEPA University Hospital, Thessaloniki, Greece; 2 Department of Oral Surgery and Radiology, School of Dentistry, Aristotle University, Thessaloniki, 541 24, Greece; 3 Department of Head and Neck Surgery, Barnet and Chase Farm Hospitals NHS Trust, London, UK; 4 Department of Pathology, Aristotle University of Thessaloniki, Thessaloniki, Greece; 5 Otorhinolaryngology, Head & Neck Surgery Department, Papanikolaou General Hospital of Thessaloniki, Thessaloniki, Greece

## Abstract

Dermatofibrosarcoma protuberans (DFSP) is a rare cutaneous neoplasm associated with a high cure rate. We present a case of aggressive DFSP with fibrosarcomatous areas in the head and neck. A 28-year-old Mediterranean female presented with a 45-day history of rapidly growing cutaneous lesion of the face. Surgical biopsy confirmed the diagnosis of DFSP. Subsequently, the patient underwent wide local surgical resection, followed by reconstruction. Histopathology report revealed fibrosarcomatous transformation and the patient underwent adjuvant radiotherapy. The patient continues to be disease free at the 35-month follow-up.

Although DFSP behave as non-aggressive malignancy, surgery with complete removal of the affected area is the intervention of choice. Moreover, adjuvant treatment and follow-up of the patient is essential in order to prevent recurrence.

## Introduction

Dermatofibrosarcoma protuberans (DFSP) is a locally aggressive, cutaneous, malignant tumor characterized by high propensity for local relapse and low metastatic potential. It was first recognized by Taylor [1] in 1890, and described by Darrier [2] in 1924, but the term "dermatofibrosarcoma protuberans" was coined by Hoffman [3] in 1925. It has been reported to involve many body surfaces, mainly the trunk (42-72%), followed by the extremities (16-30%) and less commonly in the head and neck (10-16%) [4]. Although it constitutes less than 0.1% of all malignant neoplasms, it represents the most frequent skin sarcoma (nearly 1% of all soft tissue sarcomas), more than 1% of all head and neck malignant tumours and 7% of all head and neck sarcomas [5,6].

Approximately 85-90% of all DFSPs represent low-grade tumours. The remaining 10-15% contains a component of high-grade fibrosarcoma. This transformation, presenting in more than 5% of tumour volume, is characterized by a higher incidence of local relapse and distance metastasis.

The characteristic villous pattern of extension into the subcutaneous fat, fascia and muscles and at the same time preservation of healthy tissue from resection represents a surgical challenge, as failure of complete excision leads to local recurrence. We report a rare case of an aggressive head and neck DFSP with fibrosarcomatous areas. We also discuss the epidemiology, clinical and pathologic characteristics and treatment.

## Case report

**Figure 1:**
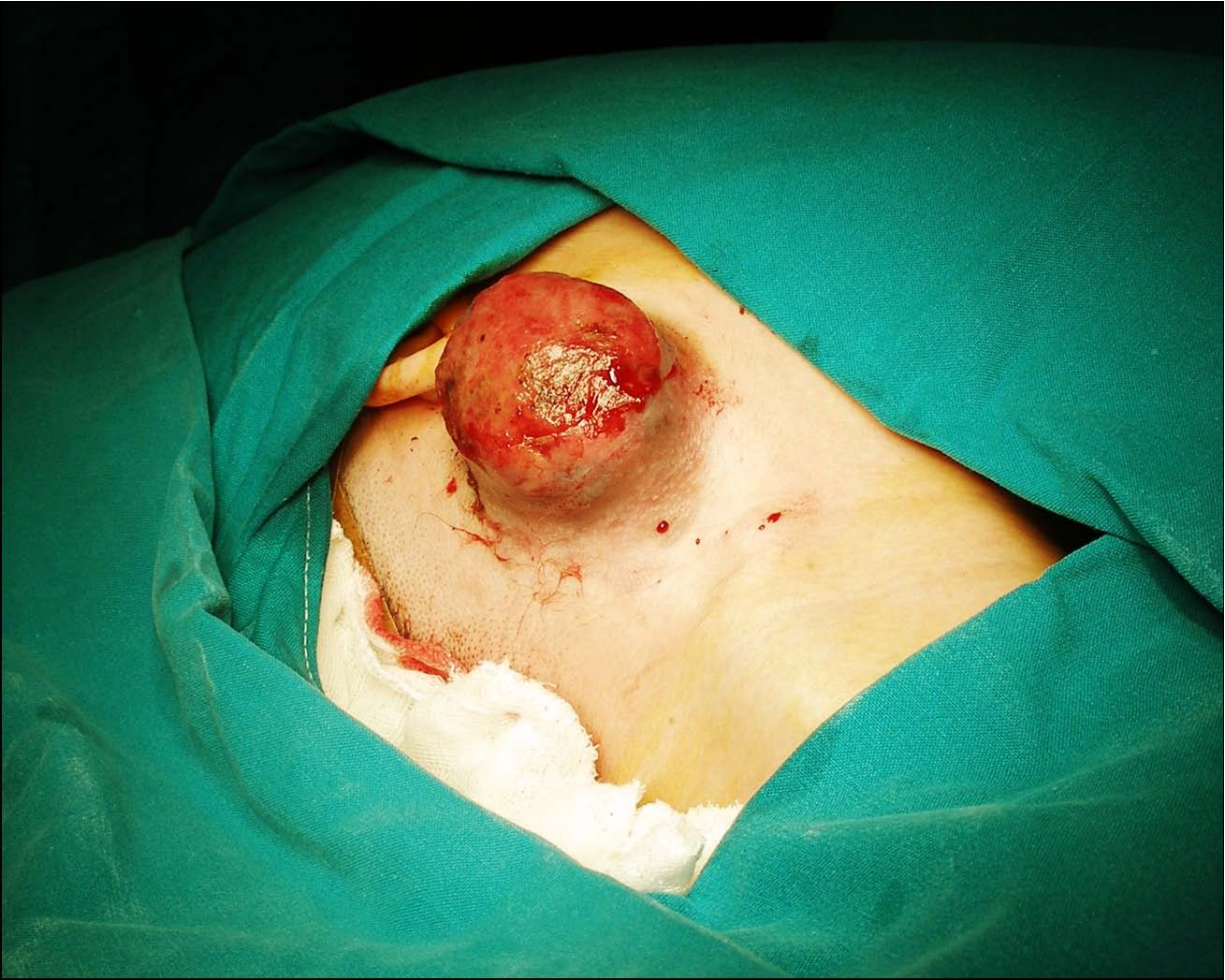
**Clinical image showing an infra-auricular lesion (10 × 5 × 4 cm in size)**.

A 28-year-old Mediterranean female with unremarkable medical history, attended the out-patients department, with a 45-day history of a painless rapidly growing lesion in the right cheek (infra-auricular area). Clinical examination revealed a protruding painless red-bluish mass, which was associated with the skin over the parotid area. The lesion (sized 10 × 5 × 4 cm) was firm, fixed to deeper tissues with propensity to bleed. No facial nerve involvement was noted (Figure 1).

**Figure 2:**
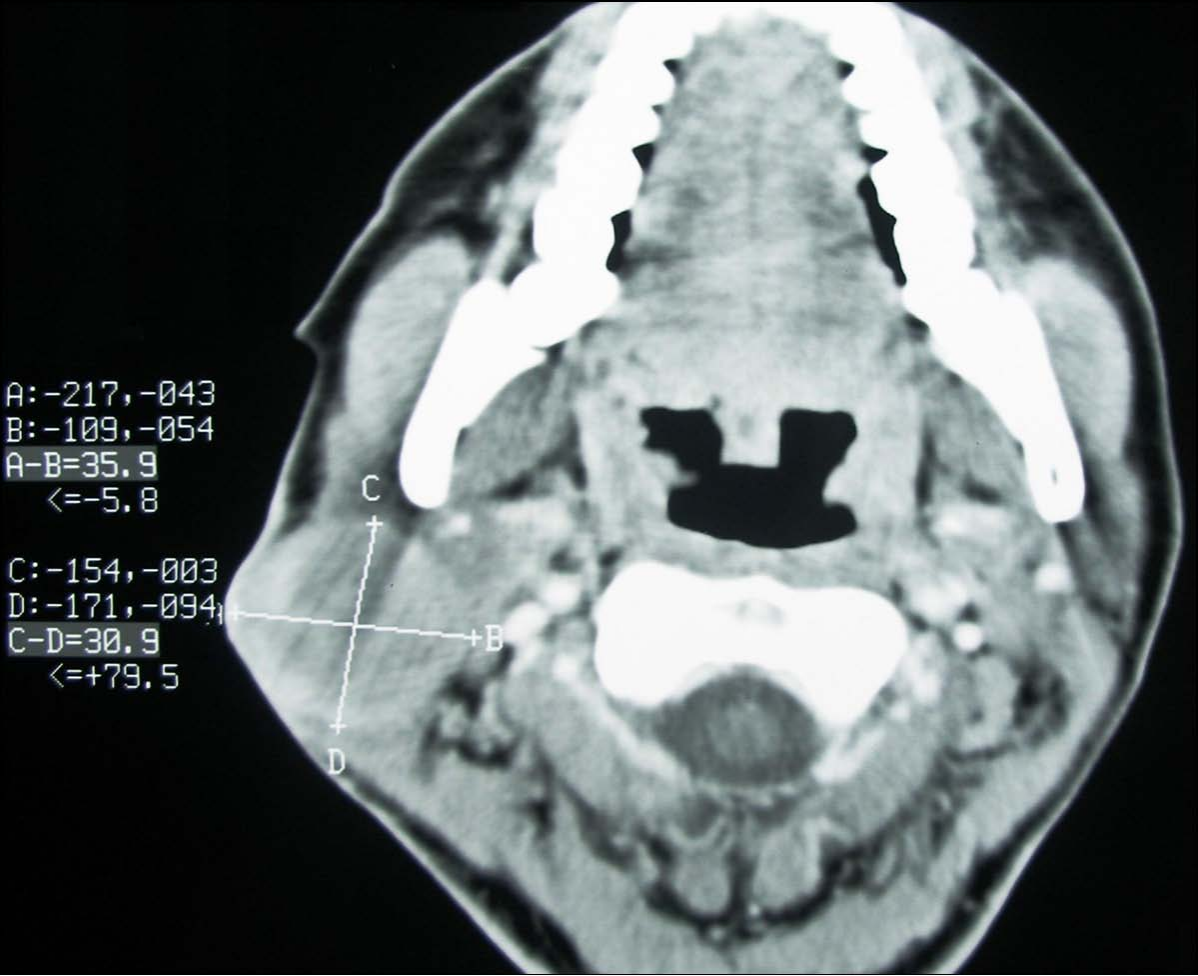
**Computed tomography image showing a parotid mass adherent to overlying skin**.

**Figure 3:**
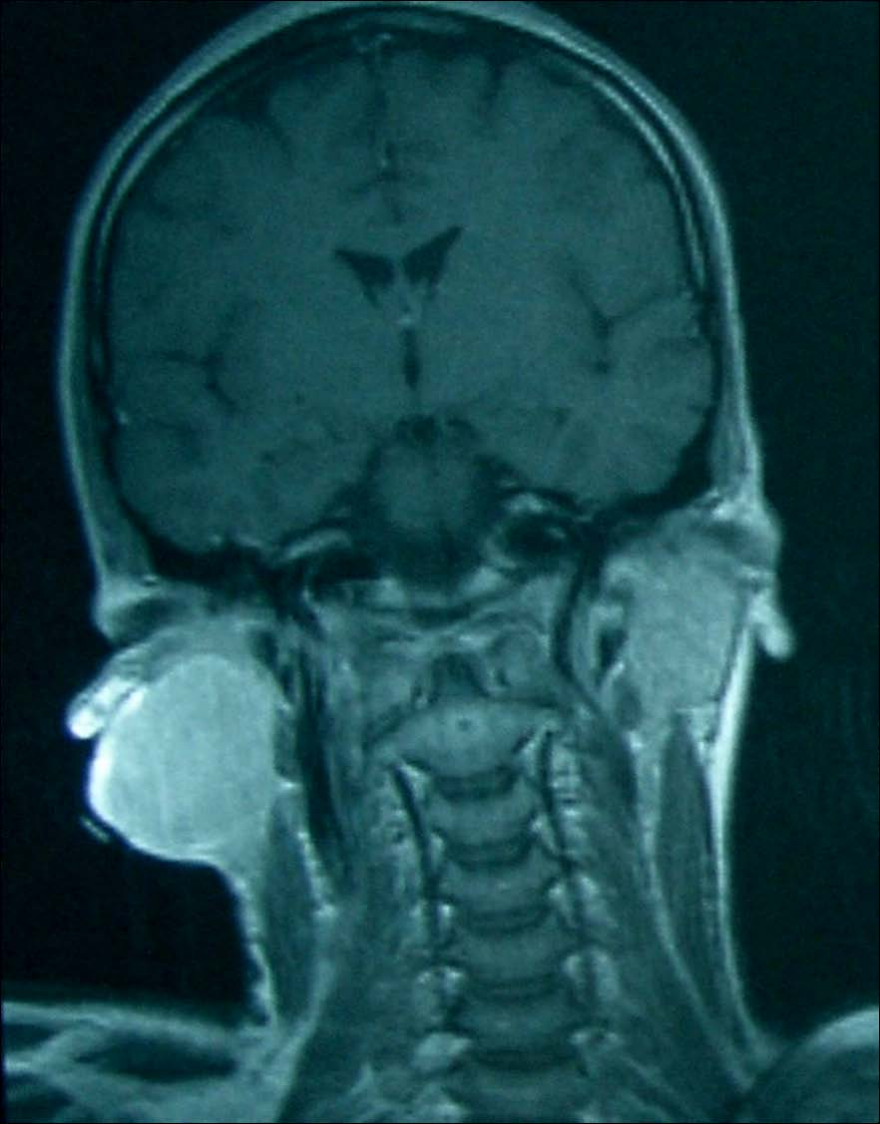
**Magnetic resonance image showing a soft tissue mass associated with the parotid gland**.

Ultrasonographic examination revealed a well vascularized mass infiltrating the subcutaneous fat and the parotid. Computed tomographic and magnetic resonance imaging evaluation revealed a parotid mass fixed to sternocleidomastoid muscle and adherent to overlying skin; multiple small lymph nodes involvement along the jugular vein were also noted (Figures 2 and 3). No distant metastasis was reported. Fine needle aspiration cytology reported a mesenchymal lesion; incisional (true-cut) biopsy, under local anesthesia showed a possible grade II sarcoma (storiform malignant fibrous histiocytoma).

**Figure 4:**
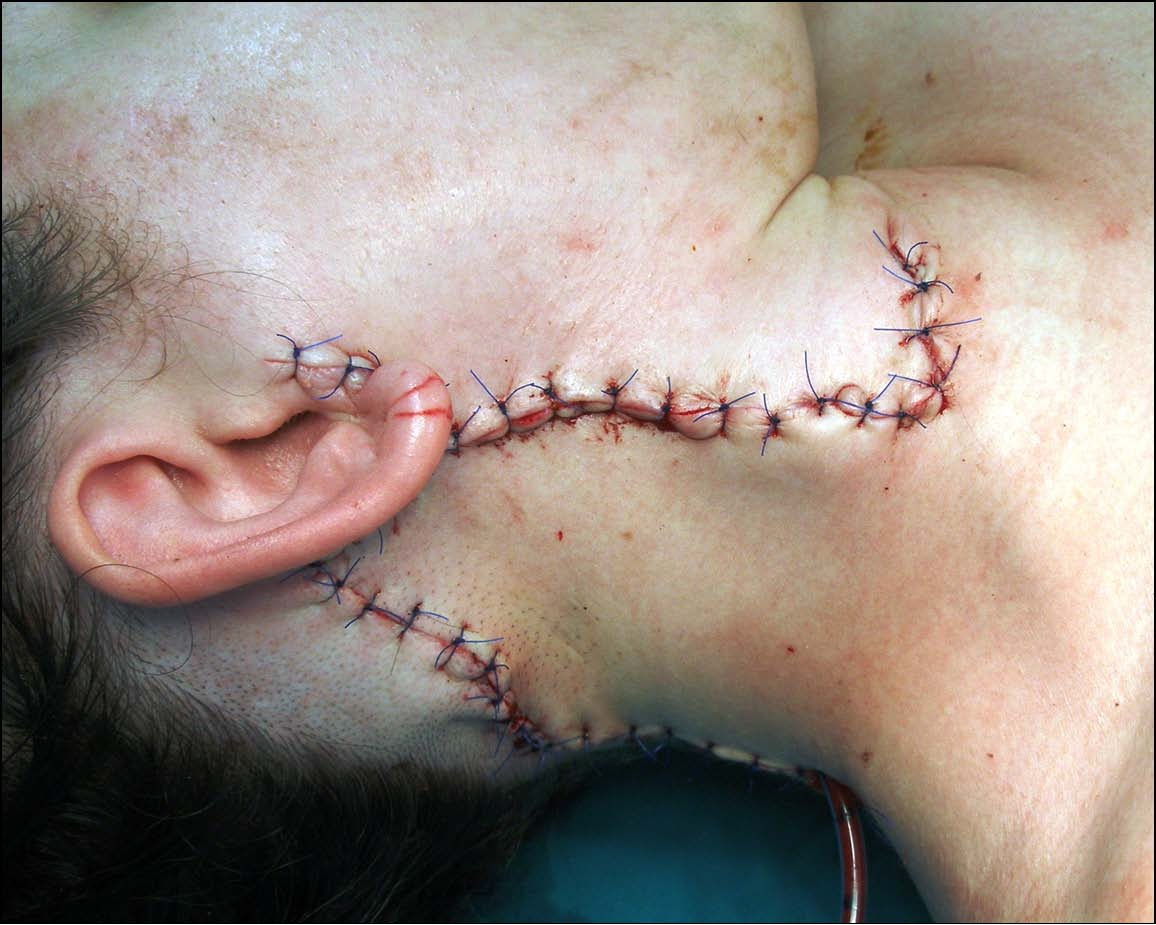
**Postoperative image following local excision and reconstruction**.

The decision was made, in a multi-disciplinary meeting, to treat the tumour with wide local resection. This involved a superficial parotidectomy and selective neck dissection (levels II-V). The defect was reconstructed with a large neck pedicle advancement flap (Figure 4).

**Figure 5:**
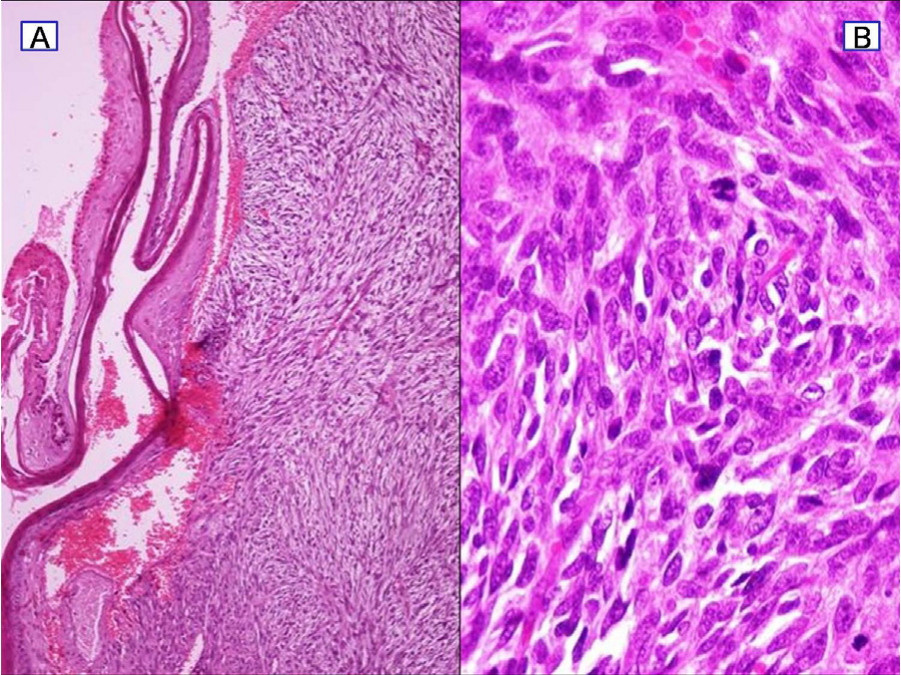
**Histopathology image showing dermatofibrosarcoma protuberans**. (a) H&E ×10 spindle-shaped tumor cells in a "cartwheel" pattern. (b) H&E ×40 showing increase number of mitosis.

**Figure 6:**
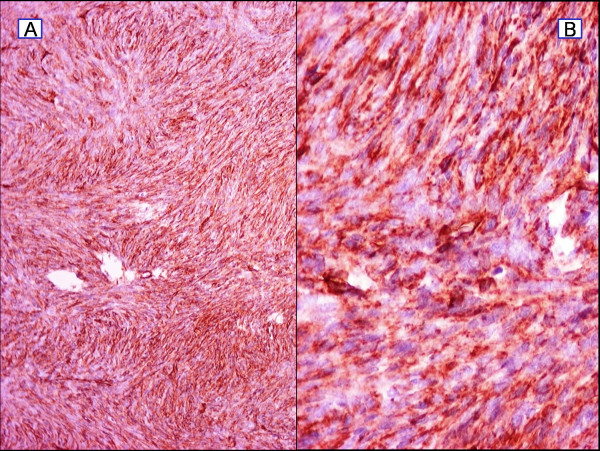
**Histopathology image showing immunopositivity to CD34**. (a) ×25 showing immunopositivity to CD34. (b) ×40 showing immunopositivity to CD34.

Histopathological examination, of the completely excised tumour, revealed a nodular neoplastic lesion composed of large spindle-shaped cells with prominent nuclei, showing a high mitotic index and areas of necrosis, infiltrating into the subcutaneous fat and muscles (Figure 5). Further immunohistochemical analysis revealed tumour cells which were positive for CD34, and c-kit antigen. A few cells were positive for SMA and CD68. Staining for Ki67/MIB 1 showed 50% positive cells and increased mitotic rate (>20/10 HPFs). It was concluded that this constellation of staining represented a "dermatofibrosarcoma protuberans with fibrosarcomatous transformation" (Figure 6).

Following the diagnosis, the multi-disciplinary team decided that an adjuvant therapy would be required. The patient, subsequently, received 60 Gy of radiation therapy and subsequent recovery was unremarkable. At 35-month post-treatment follow-up, the patient continues to be symptom free with no signs of tumour recurrence.

## Discussion

### Epidemiology

The estimated incidence of DFSP is 4.5 cases per million persons per year in the USA [7], nearly 3 in France [8] and 4 in Sweden [9]. In some studies, a slight male predominance (55-57%) [10,11] has been reported, although in others no gender predilation was established [7]. The incidence among Afroamericans compared to Caucasians is almost double (6.5 vs 3.9 per million) [12]. It affects almost every age. Although, it appears predominantly in adults (20-50 years) it has also been reported in children [13-16]. No evidence of hereditary or familial predisposition exists. The 5-year relative survival rates for reported in all population-based studies are can reach up to 100% [7,8,12].

### Macroscopically

DFSP is a dermis origin cutaneus neoplasm characterized by slow infiltrative growth with a tendency for local relapse, after surgical excision, with little metastatic potential. Clinically the appearance of the tumour depends on the stage of the disease. Initially it presents as a cutaneous pink to red-bluish painless trophic and/or sclerotic plaque-like mass that develops into lumpy nodular and over time into ulcerative hemorrhagic protuberant tumor. It develops superficially, mobile upon palpation as it is adhered with its overlying skin, but not with its underlying tissues. Unfortunately, fixation to deeper structures such as fascia and muscle may present in the later stage of the tumour. Scalp fixation caused by periosteal attachment may occur in early stages. Telangiectasia may be apparent on the surface or at the periphery.

Since it is a slow growing tumor, the duration of development range from weeks to years. Delay in diagnosis and clinical misdiagnosis of the initial lesion is not uncommon, and is due to absence of symptoms. Pain and tenderness are rare, as only 10-25% of the patients reported these symptoms. In addition cachexia which usually characterizes advanced malignancies is also uncommon [5]. Differential diagnosis in the initial stages should include lipomas, epidermal cysts, keloid and nodular fasciitis. In late stages, when it becomes protuberant it should be differentiated from pyogenic granuloma and other soft tissue sarcomas.

### Histopathology

The true cellular origin of this neoplasm is yet unclear. Evidence exists that its origin may be fibroblastic, neuroectodermal, histiocytic or from pluropotential progenitor cells that have the capacity to differentiate into these three cell-types. Microscopically it is characterized by the arrangement of spindle-shaped tumor cells in a "cartwheel" pattern [17], cytologically monomorhous bland spindle cells, with a characteristic finger-like, honeycomb pattern of infiltration into the subcutaneous fat. These neoplastic projections, like pseudopodia may eject up to 3 cm peripherically.

Immunohistochemical staining demonstrates strong positivity for CD34 (sensitivity 84-100%) and vimentin and negativity for S-100, factor XIIIa and CD44 staining. Positivity of this last marker and stromelycin 3 (ST3) is useful for differential diagnosis of benign fibrous histiocytoma (dermatofibroma) [18]. Apolipoprotein D has also been described as a marker for DFSP, as West et al. [19] in 2004 concluded, that it is strongly expressed in DFSPs and neural lesions and may be useful in differentiating DFSP from benign fibrous histiocytoma (dermatofibroma). The differential diagnosis should also include malignant fibrous histiocytoma, atypical fibroxanthoma, diffuse neurofibroma, giant cell fibroblastoma, myxoid liposarcoma, myxofibrosarcoma and desmoplastic melanoma [5].

Bendar or pigmented DFSP represents an unusual variant distinguished by the dispersal of melanin-containing dendritic cells in an otherwise typical DFSP. It accounts less than 5% of all DFSP cases predominantly occur in Afroamericans (7.5 times higher than Caucasians). Other unusual histologic types include myxoid, giant cell angiofibroma, granular cell variant DFSP, palisaded (reminiscent of schwannoma), sclerosing and the atrophic type.

A more aggressive subtype presents with fibrosarcomatous progression is called "fibrosarcomatous dermatofibrosarcoma" (FS-DFSP). Firstly reported by Penner [20] in 1951 to represent a component of intermediate to high grade sarcoma, with more spindle cells, greater number of nuclei and increased mitotic rate compared to "classic" DFSP(median: 20 vs. 2 mitoses/HPFs), immunohistochemically demonstrating a decrease of CD34. It is regarded as a rare lesion with approximately 50 cases reported until 1998 [21]. It appears in approximately 10-15% of DFSP cases, characterized by a higher incidence of local relapse and distance metastasis. According to Mentzel et al. [21] progression of DFSP to FS-DFSP may represent "dedifferentiation" and Abbott et al. [22] demonstrated that "FS change in DFSP represents a form of tumor progression with increased risk of metastasis over classic DFSP, associated with gains of p53 mutations and increased proliferative activity".

### Molecular Pathogenesis

Although an injury to the affected skin, such as surgical and old burn scars and sites of vaccinations may be predisposing factors for PDFS development [17,23], its cause is yet unknown. More than 90% of DFSP are characterized by reciprocal chromosomal translocation between chromosomes 17 and 22, t(17;22) or supernumerary ring chromosomes composed of interspersed sequences from bands 17(17q22) and 22(22q12). It is now clear that this fusion is the main abnormality in its molecular identity. This chromosome rearrangement fuses the strongly expressed collagen 1-Alpha-1gene (COL1A1) on chromosome 17 with the platelet derived growth factor (PDGF-B) gene on chromosome 22. PDGF-B, which is a potent mitogen for connective tissue cells, is placed under the control of COL1A1 promoter. This results in autocrine activation of the platelet derived growth factor receptor tyrosine kinase (PDGF-R) which triggers the proliferation of DFSP tumor cells.

### Staging

Since clinical diagnosis of the initial lesion is not always possible with certainty, open biopsy, excisional or incisional, is the diagnostic method of choice. Imaging using MRI to evaluate local extension is essential for the preoperative planning of large tumors and computed tomography is useful only when underlying bone erosion is suspected. Ultrasonographic examination may also be useful, mainly in detecting small lesions. Although lymphatic and hematogenous metastasis is uncommon, staging is always indicated.

The American Joint Committee on Cancer has not yet set a system for the staging of DFSPs and DFSP-FSs. Until today it is in accordance with the American Musculoskeletal Tumor Society (MSTS) staging system, which takes into account tumor grade and compartmentalization: In stage IA tumors are low-grade intracompartmental (without extension beyond the subcutaneous compartment) lesions, that can be managed adequately solely with wide excision. In stage IB tumors are again low-grade lesions that exhibit extracompartmental extension, which involves the underlying fascia, muscle, or bone erosion [24]. More recently, Ugurel et al. [25] proposed a staging system according to German Guidelines for DFSP. In this system stage I represent the primary tumor stadium, stage II describes a DFSP with regional lymph node metastases and stage III characterizes distance metastases.

### Treatment and Prognosis

DFSPs show an extremely aggressive tendency to invade local surrounding tissue. Standard therapeutic approach used for the treatment of this tumor is wide and deep local excision (WLE), including the underlying fascia. In a retrospective study of 159 cases, although 99% of the cohort had complete surgical resection, pathologic review showed only 93 patients (58%) with negative microscopic margins [11]. Lindner et al. [26] reported that 2.5-3.5 cm circumferential removal of healthy tissues improved local control of the disease. It is yet generally agreed that 3-5 cm lateral and deep margins are adequate for the local control of the disease.

In order to achieve negative resection margins and simultaneously preserve the uninvolved tissue from resection, some authors suggest the use of Moh's micrographic surgery (MMS). This method allows precise histological mapping of all margins, both deep and lateral. Classic technique requires continuing sequential horizontal sectioning during resection and immediate, frozen, microscopic examination, until free margin is obtained. Although ideal, especially for challenging areas such as head and neck region, it may be proven inadequate predictor of final resection margin status. A study involving intraoperative assessment of frozen sections concluded that accuracy was low - 80% (16 out of 20) [6]. On the other hand the modified method, using paraffin-embedded sections, is a more accurate but at the same time, a very elaborated and time-consuming. The largest single centre published series on outcomes of DFSP, demonstrated that WLE with reconstruction can give disease control in nearly 90% of the cases [27]. In contrast to Paradisi et al. [28] in a study of 79 patients treated with WLE (n = 38) or MMS (n = 41) between 1990-2005 established 13.2% local recurrence rate (5/38 patients, 95% CI 4.4-28.1%, follow-up of 4.8 years) and none (95% CI 0-8.6%, follow-up of 5.4 years), respectively. In the same study a review of the literature yielded 6/463 recurrences for MMS (1.3%, 95% CI 0.5-2.8%) and 288/1394 recurrences for WLE (20.7%, 95% CI 18.6-22.9%).

Since primary closure is not always feasible, reconstructive surgery, using local skin flap, skin grafting, mesh or myocutaneus flap may be required. Neck dissection is not necessary unless suspicious regional lymphadenopathy is present. Only in FS-DFSP cases sentinel lymphnode biopsy is recommended.

#### Local Recurrence

The most significant prognostic factor for relapse has proved to be the extent of the initial resection as close margins (<2 cm) shows a statistically significant positive correlation with recurrence. Rutgers et al. [10] in an extensive review of 913 DFSP cases in the literature reported nearly 50% overall recurrence rate decreasing to 13% after adequate wide excision. Lemm et al. [4] in their review of the literature established a 39.7% total recurrence rate in 116 patients with undefined or conservative surgical margins. In the same review the total recurrence rate of 661 patients underwent WLE was decreased to 8.8%. In a similar analysis of the literature, Gloster et al. [29] reported 43% recurrence rate in a review of 317 patients and 18% in a review of 489 patients respectively. Moreover, in the largest monoinstitutional series of DFSP, in Italy, all 218 patients were treated with WLE and reconstructive surgery, 10 year local relapse incidence was 4% [27]. In contrast, a recent study of 204 patients treated with WLE with relatively narrow margins of 1-2 cm, established an incidence of 5-year local relapse of only 1%, recognizing that additional follow-up may increase it [30].

The Head and neck region is reported to be the site with the highest rate of local recurrence rate (LRR) after local excision. Barnes et al. [31] reported 17 personal cases of head and neck DFSP (HN-DFSP) with 53% LRR. Same authors in a review of the literature established 73% LRR in a series of 92 patients. Mark et al. [32] also reported very high LRR (60%) in a series of 16 patients suffering HN-DFSP. Although Stojadinovic et al. [6] reported a "normal" LLR of 9% in their series of 33 patients with HN-DFSP, 12 of these patients (36%) presented in their centre with already recurrent disease, after prior local excision elsewhere. In addition, in Farma's [30] recent study of 204 DFSP cases, the only two local recurrences reported in the head and neck. It is understood that head and neck surgeons are more conservative due to the critical structures of the area and the cosmetic difficulties in reconstruction of the surgical defect. On the other hand multiple recurrences caused by inadequate control of the initial disease predisposes to distant metastasis and poor outcome [10].

FS-DFSP subtype is also considered to be a highly significant prognostic factor for relapse. In a large prospective analysis of 159 patients, 13 of 25 patients (52%) with FS-DFSP experienced relapse versus 21 of 134 patients (16%) with classic DFSP. In the same analysis the 5-year recurrence free survival rates were 81% for patients with DFSP and only 28% for those with FS-DFSP subtype [11]. In addition in Mentzels study [21], follow up of 34 from 41 FS-DFSP patients revealed local relapse in 20 patients (58%). Only Goldblum et al. [33] and Abbott et al. [22] referred lower LLR (22% and 20%) in a series of 17 and 41 FS-DFSP cases respectively.

Other unfavorable prognostic factors for local and distance relapse, represents age older than 50 years, high mitotic rates and increased cellularity. Regional lymphatic metastasis is uncommon, approximately 1%, even less frequent than distant metastasis with only a few cases reported in the literature up to date [10,34].

#### Distance Metastasis

Although very rare, DFSP do metastasize, principally to the lungs and bones. Rutgers et al. [10] reported 37 metastases (4%) in a review of 913 cases in the literature. Earlier Das Gupta [35] reported 27 cases (5.7%) after reviewing 475 cases in the literature. In contrast Bowne et al. [11] referred a metastatic rate of only 1% (2 out 159 patients). Interestingly these 2 patients, both succumbed to their disease, suffered from FS-DFSP, representing 8% (2 out of 25 patients) metastatic rate in this subtype. Also in the largest monoinstitutional series of 218 patients suffering DFSP (211 classic and 7 FS-DFSP) treated with wide local excision, the incidence of 10 year metastasis was low (2%, 5 patients). Again from the 7 patients suffering FS-DFSP, 2 (28%) developed and died from pulmonary, soft tissue and osseous metastasis, one of them without prior local recurrence [27]. Furthermore Ding et al. [36] focused on FS-DFSP tumours, reviewed 21 cases and established an overall metastatic rate of 14.3% (3 patients). In addition Menzel et al. [21] reported similar metastatic rate (14.7%, 5 out of 41 FS-DFSP patients), all pulmonary, one with additional soft tissue and one with additional multiple osseous metastases. Two of these patients (5.8%) died from disease progression. Same author in a review of the literature reported another 6 cases (13%) of metastases and 11% of tumour-related death concluding that FS-DFSP should be designated as a fully malignant soft tissue neoplasm in contrast with classic DFSP. A more recent series of FS-DFSP from a single institution also evaluated high metastatic rate (10%, 4 out of 41 patients) [22]. In this study one patient experienced pulmonary and osseous metastases, 2 pulmonary and one osseous metastasis. Two of these patients (5%) succumbed to their disease. In contrast Goldblum et al. [33] in their analysis of 17 cases of FS-DFSP with at least five years follow-up reported no metastatic disease in patients treated with wide local excision.

#### Conservative Treatment

In the past, radiotherapy (RT) was considered to have limited role in treatment of this disease. Recently published data demonstrated that DFSP is a radiosensitive tumour. An analysis of 10 cases, 9 DFSP and 1 FS-DFSP, established 90% local control of the disease, concluding that adjuvant radiotherapy reduces the risk of local relapse after resection of the disease in close or positive margins. Interestingly, 60% (6 patients) of these cases were located in the head & neck and the patient suffering from FS-DFSP, also located in head and neck, was the one who experienced the local recurrence after 3 months and died from the disease [37]. Similarly Sun et al. [38] in a series of 35 patients, 24 treated surgically and 11 with surgery and RT, reported 7-year local control rates of 28% and 80% respectively.

Preferably the excision should followed by adjuvant radiotherapy, when margins are found close or persistently positive and repeat wider resection is not feasible due to anatomic limitations. Some authors also recommend the use of radiotherapy after wide resection in the fibrosarcomatous subtype, even with negative margins [30]. German guidelines for DFSP treatment suggests 60 and 70 Gy for micro- and macroscopic disease, respectively, including primary tumor, postoperative scars and a safety margin of 3-5 cm and palliative dosage of 50 Gy [25].

Although chemotherapy is proved to be ineffective, recently targeted therapy has shown very good results in disseminated cases. Imatinib mesylate, a selective tyrosine kinase inhibitor designed to treat chronic myelogenous leukaimia, resulted also in inhibiting PDGFR tyrosine kinase that plays a crucial role in the pathogenesis and tumor growth of DFSP cells.

A phase II study published in 2005, reported 100% response in 8 local advanced DFSPs, partial response in a metastatic FS-DFSP, all with t(17;22) translocation and no clinical response in another metastatic FS-DFSP lacking t(17;22), correlating the presence of this translocation with response. All patients were treated with 400 mg of imitinib twice daily, well tolerated, decreasing dosage into 600 mg in only one patient [39]. It is generally accepted that the use of this drug is indicated in patients with unresectable, locally advanced, recurrent or metastatic disease. It can also be used in tumor shrinkage prior to surgery, avoiding loss of functions and cosmetic defects. A recent study reported tumour size reduction and decreased cellularity after preoperative treatment with imatinib [40]. More studies are needed to determine whether it could play a role in neoadjuvant setting.

## Conclusion

DFSP is a rare dermal malignancy with a propensity to be locally aggressive but rarely metastatic. Its fibrosarcomatous progression variant on the other hand has a more aggressive course in nature, with a significant elevated risk of both local and distance metastasis, usually followed by poor outcome.

Wide local excision is the gold standard treatment and a policy of re-excision to obtain negative margins should always be followed. One of the most challenging areas is the head and neck, with increased rate of local failure, due to critical structures and aesthetic difficulties in reconstruction.

We reported a case of an aggressive head and neck FS-DFSP, treated with wide local resection and adjuvant radiotherapy, without evidence of local or distance relapse after 35 months. Follow-up, even lifelong, remain essential.

## Consent

Written informed consent was obtained from the patient for publication of the case report in this review and accompanying images. A copy of the written consent is available for review by the Editor-in-Chief of this journal.

## Competing interests

The authors declare that they have no competing interests.

## Authors' contributions

NA, PK, WJ, TU, ST, GK, AN conceived of the study, and participated in its design and coordination. All authors read and approved the final manuscript.
